# Comparison of the diagnostic accuracy of contrast-enhanced/DWI MRI and ultrasonography in the differentiation between benign and malignant myometrial tumors

**DOI:** 10.1016/j.amsu.2021.102813

**Published:** 2021-09-21

**Authors:** Shaparak Najibi, Mitra Modares Gilani, Fatemeh Zamani, Setare Akhavan, Narges Zamani

**Affiliations:** a Department of Obstetrics and Gynecology, Vali-Asr Hospital, Tehran University of Medical Sciences, Tehran, Iran; b Department of Gynecologic Oncology, Vali-Asr Hospital, Tehran University of Medical Sciences, Tehran, Iran; c Department of Radiology, Children Medical Center of Excellence, Tehran University of Medical Sciences, Tehran, Iran

**Keywords:** Malignant myometrial tumors, MRI, Ultrasonography, Cross sectional, Diagnostic value, Test accuracy

## Abstract

**Background:**

Various modalities including ultrasonography and magnetic resonance imaging (MRI) have been developed as imaging technique for screening malignant myometrial tumors, but a few studies assessed the diagnostic value of these two techniques in differentiation of benign from malignant myometrial tumors that had been the main purpose of this study.

**Materials and methods:**

This cross-sectional study was performed on 63 women underwent surgery for intrauterine masses that were initially assessed using MRI and ultrasound before surgery at a tertiary hospital in Tehran from 2016 to 2020. Their MRI was reviewed by a reputable radiologist in the field. The findings of histopathological assessment were considered as the gold diagnostic standard.

**Results:**

The sensitivity, specificity, positive predictive value (PPV), negative predictive value (NPV), and accuracy of MRI to detect sarcoma were revealed to be 94.6%, 92.3%, 94.6%, 92.3%, and 93.7% respectively. Ultrasonography had not preferable applicability to differentiate sarcoma from benign tumors with sensitivity, specificity, PPV, NPV and accuracy of 35.1%, 88.4%, 81.2%, 48.9%, and 57.1% respectively. The diagnostic performance of both modalities was not affected by baseline clinical conditions including pain, abnormal uterine bleeding and menopausal status.

**Conclusion:**

MRI but not ultrasonography can effectively differentiate benign from malignant myometrial tumors.

## Highlights

• Pretreatment Diagnosis of malignant myometrial tumors may be challenging.
• MRI with DWI can be considered as a diagnostic tool in leiomyosarcoma.
• Pretreatment distinction between benign and malignant myometrial tumors is necessary.

## 1 Introduction

The assessment of myometrial tumors is one of the main indications for pelvic imaging [1]. Along with histological analysis that has been identified as the gold standard for accurate analysis of surgical mass specimens in such tumors, evaluation of various dimensions of the mass and its extension will also require evaluation through imaging [2]. In addition, the use of minimal invasive methods will lead to greater potential satisfaction of the patient and the surgeon. This becomes even more important when a hysterectomy is necessary due to the malignant nature of the mass [3]. More important, the lack of pretreatment suspicion of malignant lesions such as leiomyosarcoma may expose the affected patients to increase the likelihood of intra-peritoneal dissemination as well as distance metastases and in this regard, the demarcation between conservative treatments and invasive surgery is also difficult [4]. In return, uterus-preserving treatments such as hormone therapies or uterine arterial embolisation in benign tumors should be considered [5]. Therefore, the distinction between benign and malignant masses in cases of suspected myometrial masses is not only necessary in determining the best treatment regimen but also in providing the optimal prognosis of treatment and in this regard, imaging methods are in the forefront.

Ultrasonography has been the first-line imaging technique for assessment of myometrial tumors; however, its partially low diagnostic performance in detection of heterogeneous solitary tumors with high vascularity has been shown in several studies [6].

CT plays a limited role in the initial diagnosis and local staging of myometrial lesions. CT is excellent for demonstrating calcifications; they are often found in leiomyomas but may also be present in LMSs [7].

In this regard, the application of magnetic resonance imaging (MRI) as the second line imaging tool for characterization of such tumors has been considered particularly in those suspected large masses with high T2-weighted signal intensity [8]. Nevertheless, the technique will always be preferred when is able to discriminate the masses with different degeneration degrees or cellular histological subtypes and ultimately differentiate benign from malignant natures. However, a few studies assessed the diagnostic value of ultrasonography and MRI in differentiation of benign from malignant myometrial tumors that had been the main purpose of this study.

## 2 Materials and methods

### 2.1 Registration and ethical approval

This study was conducted in accordance with the Helsinki Declaration and was approved by the Tehran University of Medical Sciences ethics committee (IR.TUMS.IKHC.REC.1398.110) [9].

### 2.2 Study design

This cross-sectional study was performed on women who underwent surgery for intrauterine masses who assessed using MRI with and without contrast as well as DWI and ultrasound before surgery at a tertiary hospital in Tehran from 2016 to 2020.

### 2.3 Sample

The sampling method in our study was census and patients were selected from the available samples.

### 2.4 Procedure/protocol

In this study, an experienced radiologist in gynecological radiology reported MRI images without knowing the patient's clinical symptoms and histopathology. The lesion with largest size was examined in the case of multiple uterine's tumor. MRI performed with 1.5 T GE machine. Pelvic phased array coils were used. Axial, central and sagittal T2w series of the pelvic and additional true axial and sagittal T2w of uterus body were performed. Axial T1w pre-contrast imaging and dynamic contrast enhanced imaging were also used. In all patients diffusion weighted imaging was routinely obtained. DWI was considered restricted when it had more than 50% of the signal above the external myometrium at the high b-value DWI (b = 1000 s/mm2). Evaluation of the features and nature of the mass by ultrasonography (Transabdominal &Transvaginal grayscale and color Doppler) was also considered which was done by different sonographers in our center, however, all MRI performed with suitable protocol were reviewed by another reputable radiologist. In heterogeneous masses with central necrosis, color doppler ultrasound was shown increased velocity. Also, the reference standard used in this study was histopathology, a surgical specimen that was examined by an experienced pathologist in the field of gynecological pathology. Histopathological diagnosis was based on criteria proposed by Bell et al. [10], namely coagulation necrosis, cellular atypia, cellularity, and the number of mitotic forms in 10 high-power fields (HPF) on H&E-stained slides. The study endpoint was to determine the diagnostic value of ultrasonography and MRI in differentiation of benign from malignant myometrial tumors by considering histological assessment as the gold diagnostic standard.

### 2.5 Data collection

In this study, in order to collect data, the study checklist which includes baseline characteristics, clinical data, and laboratory parameters as well as information about imaging findings was used.

### 2.6 Statistical analysis

For statistical analysis, results were presented as mean ± standard deviation (SD) for quantitative variables and were summarized by frequency (percentage) for categorical variables. Continuous variables were compared using *t*-test or Mann-Whitney test whenever the data did not appear to have normal distribution or when the assumption of equal variances was violated across the study groups and qualitative variables were analyzed with chi-square test. The degree of agreement between the diagnostic findings between the two techniques was determined and evaluated based on the kappa agreement coefficient (Table 3). Also, in determining the diagnostic value of each technique in comparison with the gold standard, the calculation of diagnostic indicators including sensitivity, specificity, positive predictive value (PPV), negative predictive value (NPV) and accuracy was considered. P values of ≤0.05 were considered statistically significant. For the statistical analysis, the statistical software SPSS version 23.0 for windows (IBM, Armonk, New York) was used.

**Table 3:** The association of pathological results with ultrasonography according to baseline parameters.

	Pathological malignancy (+)	Pathological malignancy (−)	Kappa value
Before menopause			0.276
Positive malignancy	10 (76.9%)	3 (23.1%)	
Negative malignancy	18 (46.2%)	21 (53.8%)	
After menopause			0.299
Positive malignancy	3 (100%)	0 (0.0%)	
Negative malignancy	6 (75.0%)	2 (25.0%)	
With pain			0.265
Positive malignancy	8 (80.0%)	2 (20.0%)	
Negative malignancy	11 (52.4%)	10 (47.6%)	
Without pain			0.260
Positive malignancy	5 (83.3%)	1 (16.7%)	
Negative malignancy	13 (50.0%)	13 (50.0%)	
With bleeding			0.115
Positive malignancy	5 (62.5%)	3 (37.5%)	
Negative malignancy	15 (48.4%)	16 (51.6%)	
Without bleeding			
Positive malignancy	8 (100%)	0 (0.0%)	
Negative malignancy	9 (56.3%)	7 (43.8%)	

This study is fully compliant with the STROCSS criteria www.strocssguideline.com [11].

## 3 Results

In this study, of 63 patients included into the study, 20 cases (31.7%) underwent myomectomy and 43 cases (68.3%) underwent hysterectomy. MRI assessment led to final diagnosis of sarcoma in 58.7%, while diagnosis of sarcoma was finalized by ultrasonography in 25.4%. In this regard, histological assessment also resulted in diagnosis of sarcoma in 58.7%. As shown in Table 1, concurrently diagnosis of sarcoma in both MRI and histological findings was indicted in 35 patients whereas lack of evidence of sarcoma in both diagnostic methods was reported in 24 cases. In this regard, the sensitivity, specificity, PPV, NPV and accuracy of MRI to detect sarcoma were revealed to be 94.6%, 92.3%, 94.6%, 92.3%, and 93.7% respectively. In similar analysis and considering the findings by ultrasonography, evidence for sarcoma in both ultrasonography and histological assessment was found in 13 cases, whereas negative results of both methods was reported in 23 patients yielding sensitivity, specificity, PPV, NPV and accuracy of 35.1%, 88.4%, 81.2%, 48.9%, and 57.1% respectively for ultrasonography. Regarding clinical manifestations, pain and abnormal bleeding was revealed in 49.2% and 61.9% respectively. As shown in Table 2, clinical manifestations including the presence of pain or bleeding as well as menopausal status could not affect the diagnostic performance of MRI to detect sarcoma. In this regard, the agreement between MRI and histological findings was strong adjusted for clinical manifestations. However, regarding the correlation between ultrasound findings and histological evaluation, even with the presence or absence of any clinical features including pain, bleeding, and menopausal state, the agreement between the two diagnostic methods remained still weak (Table 3).

**Table 1:** The association of pathological results with MRI and ultrasonography findings (chi-square test).

	Pathological malignancy (+)	Pathological malignancy (−)	P value
MRI			0.0001
Positive malignancy	35 (94.6%)	2 (5.4%)	
Negative malignancy	2 (7.7%)	24 (92.3%)	
Ultrasonography			0.034
Positive malignancy	13 (81.3%)	3 (18.8%)	
Negative malignancy	24 (51.1%)	23 (48.9%)

**Table 2:** The association of pathological results with MRI according to baseline parameters.

	Pathological malignancy (+)	Pathological malignancy (−)	Kappa value
Before menopause			1.000
Positive malignancy	26 (92.9%)	2 (7.1%)	
Negative malignancy	2 (8.3%)	22 (91.7%)	
After menopause			0.845
Positive malignancy	9 (100%)	0 (0.0%)	
Negative malignancy	0 (0.0%)	2 (100%)	
With pain			0.720
Positive malignancy	17 (89.5%)	2 (10.5%)	
Negative malignancy	2 (16.7%)	10 (83.3%)	
Without pain			1.000
Positive malignancy	18 (100%)	0 (0.0%)	
Negative malignancy	0 (0.0%)	14 (100%)	
With bleeding			0.950
Positive malignancy	20 (95.2%)	1 (4.8%)	
Negative malignancy	0 (0.0%)	18 (100%)	
Without bleeding			0.710
Positive malignancy	15 (93.8%	1 (6.3%)	
Negative malignancy	2 (25.0%)	6 (75.0%)	

## 4 Discussion

In the diagnosis of uterine sarcomas in general, it can be said that MRI is more useful than ultrasound or CT scan. Non-invasive diagnostic imaging has been extensively evaluated to differentiate uterine LMSs from leiomyomas given the important differences in their prognosis and management. CT plays a limited role in the initial diagnosis and local staging of myometrial lesions. CT is excellent for demonstrating calcifications; they are often found in leiomyomas but may also be present in LMSs. Despite equivocal levels of agreement over the diagnostic accuracy of individual features, MRI remains the preferred imaging modality for in-depth evaluation of myomatous uterine tumors and for delineation of local spread of malignant disease(7).

CT of the thorax, abdomen, and pelvis is widely employed preoperatively for the detection of lymph node metastases and distant spread in endometrial cancer. The primary tumors, when visible at CT, are typically depicted as slightly hypodense relative to the surrounding contrast-enhancing myometrial tissue. For local staging, CT has long been considered inferior to MRI and TVU, due to lower soft-tissue contrast resolution at CT, and recent literature reporting diagnostic performance for local staging parameters of CT is thus scarce [12].

Contrast-enhanced MRI with diffusion-weighted imaging is more accurate in the diagnosis of leyomiosarcoma and smooth muscle tumor of uncertain malignant potential(STUMP) in comparison to its accuracy in leiomyomas. MRI is currently the best diagnostic tool for preoperative examination of uterine masses to diagnose uterine leiomyosarcoma. In our study which aimed to evaluate the diagnostic power of MRI modalities in the diagnosis of leiomyosarcoma, first, we showed considerably lower diagnostic value of ultrasonography as compared to MRI in differentiation of malignant from benign tumor. In other words, the agreement between pathological assessment and MRI was found to be high, while such agreement was considerably low between pathological assessment and ultrasonography. Therefore, according to the obtained results, it can be said that MRI modality has a higher sensitivity, specificity and accuracy than ultrasound in the diagnosis of uterine leiomyosarcomas {Table 1, (0.0001 ^vs^ 0.034)}. As another finding, the value of both evaluated modalities did not affect by baseline clinical conditions including pain severity, the presence of abnormal uterine bleeding or even menopausal status. In fact, high value of MRI in detecting malignant lesions is independent to such abnormal uterine conditions, an important and practical advantage of using this tool.

Consistent with our result, Ken Tamai and colleagues in a 2008 examined the usefulness of diffusion-weight magnetic resonance imaging (DW) for the diagnosis of uterine sarcoma. The results of that study suggested that morphological features in unreinforced MR sequences and post-contrast, DW imaging and ADC measurements may have a potential ability to differentiate uterine sarcoma from benign leiomyoma [13]. In a systematic study, Helen Kaganov and colleagues in 2018 examined significant diagnostic features in MRI imaging of leiomyosarcoma. The results of this study showed that there is a significant relationship between histopathological type and T1 and T2 intensity signals [14]. In 2019, Tong et al. conducted a study to evaluate the accuracy and feasibility of performing MRI with increased contrast from the pelvis with a DWI system for leiomyosarcoma before fibroid removal. In this study, the results were consistent with our study and showed that leiomyosarcoma could be identified by this method with sensitivity of 100% and specificity of 97% [15]. Therefore, it can be concluded that MRI, especially with standard reports and coordination with treating physicians, is an effective and potentially economical screening test. In a study that was inconsistent with our study, Umesaki et al. performed a study to evaluate the effectiveness of positron emission tomography with 18F-fluorodeoxyglucose (FDG-PET) for the diagnosis of uterine sarcoma compared to the effectiveness of MRI and Doppler imaging. A comparative study was performed on the usefulness of these three diagnostic methods for the diagnosis of sarcoma. Tumors included three leiomyosarcomas, one endometrial stromal sarcoma, and one carcinosarcoma. In their survey, positivity was reported 100% for FDG-PET, 80% for MRI and only 40% for ultrasonography. In fact, it seems that FDG-PET can be the most useful diagnostic method for uterine sarcoma [16]. Overall, the use of only ultrasonography cannot preset a suitable view of tumor nature and its malignant feature.

Our study however had some potential limitation. First, the nature of the study was retrospective, thus there was a possibility that some information may be missed due to their lack in patients' files. Second, the ultrasound was performed by different people and therefore the possibility of reaching an interpersonal agreement was essentially high. In final, the small sample size of the study made it possible to reduce the study power and therefore conduct further studies with a larger sample size.

## 5 Conclusion

MRI is more efficient and applicable in differentiation of malignant from benign myometrial tumors comparing with ultrasonography. Although, ultrasonography may be recommended for initial screening of myometrial lesions.

## Ethical approval

This study was conducted in accordance with the Helsinki Declaration and was approved by the Tehran University of Medical Sciences ethics committee (IR.TUMS.IKHC.REC.1398.110).

## Funding sources

Not applicable.

## Consent for publication

All the patients signed the informed consent form. A copy of the written consent is available for review by the Editor-in-Chief of this journal on request.

## Availability of data and materials

All data generated or analyzed during this study are included in this published article [and its supplementary information files].

## Authors'contributions

SH.N: collecting, analysing and interpretation of data. M.M: final approval of the version to be submitted. F.Z: reporting and interpretation of patient's imaging. S.AKH: collecting data. N.Z: writing and editing the article, corresponding. All authors have read and approved the manuscript.

## Registration of research studies

Name of the registry: IR.TUMS.IKHC.REC

Unique Identifying number or registration ID: IR.TUMS.IKHC.REC.1398.110

Hyperlink to your specific registration (must be publicly accessible and will be checked):

https://ethics.research.ac.ir/PortalProposalList.php?code=IR.TUMS.IKHC.REC.1398.110&title=&name=&stat=&isAll=&GlobalBackPage=https%3A%2F%2Fwww.google.com%2F

## Guarantor

Narges Zamani

## Declaration of competing interest

The authors declare that they have no conflicts of interests.

## Abbreviations

MRI: Magnetic resonance imaging
PPV: positive predictive value
NPV: negative predictive value
DWI: Diffusion-weighted imaging
HPF: high-power fields
H&E-stained: hematoxylin and eosin stained
SD: standard deviation
DW: Diffusion-weighted
ADC: apparent diffusion coefficient
FDG-PET: positron emission tomography with 18F-fluorodeoxyglucose
CT: computed tomography
LMS: leiomyosarcoma
TVU: transvaginal ultrasonography
